# Palmitoylethanolamide exerts neuroprotective effects in mixed neuroglial cultures and organotypic hippocampal slices via peroxisome proliferator-activated receptor-α

**DOI:** 10.1186/1742-2094-9-49

**Published:** 2012-03-09

**Authors:** Caterina Scuderi, Marta Valenza, Claudia Stecca, Giuseppe Esposito, Maria Rosaria Carratù, Luca Steardo

**Affiliations:** 1 Department of Physiology and Pharmacology, SAPIENZA University of Rome, P.le Aldo Moro, 5-00185 Rome, Italy; 2 Department of Pharmacology and Human Physiology, University of Bari, P.zza Umberto I, 1-70121 Bari, Italy

**Keywords:** Palmitoylethanolamide, PPARα, β-amyloid, Hippocampal organotypic culture, Neuroprotection

## Abstract

**Background:**

In addition to cytotoxic mechanisms directly impacting neurons, β-amyloid (Aβ)-induced glial activation also promotes release of proinflammatory molecules that may self-perpetuate reactive gliosis and damage neighbouring neurons, thus amplifying neuropathological lesions occurring in Alzheimer's disease (AD). Palmitoylethanolamide (PEA) has been studied extensively for its anti-inflammatory, analgesic, antiepileptic and neuroprotective effects. PEA is a lipid messenger isolated from mammalian and vegetable tissues that mimics several endocannabinoid-driven actions, even though it does not bind to cannabinoid receptors. Some of its pharmacological properties are considered to be dependent on the expression of peroxisome proliferator-activated receptors-α (PPARα).

**Findings:**

In the present study, we evaluated the effect of PEA on astrocyte activation and neuronal loss in models of Aβ neurotoxicity. To this purpose, primary rat mixed neuroglial co-cultures and organotypic hippocampal slices were challenged with Aβ_1-42 _and treated with PEA in the presence or absence of MK886 or GW9662, which are selective PPARα and PPARγ antagonists, respectively. The results indicate that PEA is able to blunt Aβ-induced astrocyte activation and, subsequently, to improve neuronal survival through selective PPARα activation. The data from organotypic cultures confirm that PEA anti-inflammatory properties implicate PPARα mediation and reveal that the reduction of reactive gliosis subsequently induces a marked rebound neuroprotective effect on neurons.

**Conclusions:**

In line with our previous observations, the results of this study show that PEA treatment results in decreased numbers of infiltrating astrocytes during Aβ challenge, resulting in significant neuroprotection. PEA could thus represent a promising pharmacological tool because it is able to reduce Aβ-evoked neuroinflammation and attenuate its neurodegenerative consequences.

## Background

Alzheimer's disease (AD) is a progressive neurodegenerative disorder clinically characterized by impairment of cognitive functions and memory loss. Its two core neuropathological hallmarks are deposits of β-amyloid (Aβ) fibrils in senile plaques (SPs) and accumulation of hyperphosphorylated tau protein filaments in neurofibrillary tangles (NFTs) [1]. In vitro and in vivo findings have demonstrated that Aβ fragments promote a marked neuroinflammatory response that accounts for the synthesis of different cytokines and proinflammatory mediators [2]. After their release, proinflammatory signalling molecules act in an autocrine manner to self-perpetuate reactive gliosis and in a paracrine manner to kill neighbouring neurons, thus amplifying neuropathological damage [3]. Once considered a marginal event, appreciation of the role of inflammation in AD pathogenesis has increased rapidly in recent years [4,5]. It is believed that the inflammatory process, once initiated, may contribute independently to neural dysfunction and cell death [6]. The relevance of reactive gliosis now prompts a reconsideration of the perceived relationship between neuroinflammation and neurodegeneration, making it clear that one is not simply a culmination of the other and that both, mutually, have a crucial impact on the course of AD. On the basis of these considerations, it is now appropriate that compounds able to modulate astrocyte activation be considered as novel therapeutic tools. Among these molecules, palmitoylethanolamide (PEA) has attracted a lot of attention for its numerous pharmacological properties and its very low toxicity [7]. PEA, a naturally occurring amide of ethanolamide and palmitic acid, is a lipid messenger that mimics several endocannabinoid-driven actions, even though it does not bind to cannabinoid receptors. Converging evidence indicates that endogenous *N*-acylethanolamine compounds, including PEA, bind with relatively high affinity to peroxisome proliferator-activated receptor α (PPARα), and they are now recognized among their physiological ligands [8,9]. PPARs are a family of ligand-dependent nuclear hormone receptor transcription factors. To date, three isoforms have been identified (PPARα; PPARβ, also called δ; and PPARγ), and all three isotypes are expressed in the brain with different distributions. Although PPARβ/δ is almost ubiquitously expressed, PPARα and γ are localized to more restricted brain areas. The role of PPARs in the brain has, for the most part, been related to lipid metabolism; however, these receptors have also been implicated in neural cell differentiation and death as well as in inflammation and neurodegeneration [10,11]. PPARs stimulate gene expression by binding to peroxisome-proliferator response elements (PPREs) that are present in promoter regions of the target genes. In the absence of ligands, the heterodimers physically associate with corepressors and suppress gene transcription [12]. Upon ligand binding, the coactivators replace corepressors and activate gene expression [13].

PEA is abundant in the central nervous system (CNS), and it is conspicuously produced by glial cells [14-16]. PEA has been studied extensively for its anti-inflammatory and neuroprotective effects, mainly in models of peripheral neuropathies [17,18]. Some of its properties have been considered to be mediated by PPARα transcriptional activity [19,20]. Both PPARα and PEA are clearly detected in the CNS, and their expression may show large changes during pathological conditions [21,22]. However, its physiological role and its pharmacological properties in the CNS remain, at present and for the most part, unclear. Our group has recently demonstrated the ability of PEA to mitigate reactive gliosis induced in primary rat astrocytes exposed to Aβ by interacting with PPARα [23].

On the basis of these considerations, the present study was designed to confirm the effect of PEA on astrocyte activation in the models that we used and to verify whether its control of reactive gliosis leads to a "rebound" protection on neurons. To this purpose, our experiments were carried out using mixed neuroglia co-cultures and hippocampal organotypic slices treated with Aβ in the presence or absence of PEA. In addition, to define the molecular mechanisms responsible for the observed effects induced by PEA, further experiments were performed in the presence or absence of GW9662 and MK886, which are selective PPARγ and PPARα antagonists, respectively.

## Methods

All experiments were performed in accordance with the National Institutes of Health guidelines for the care and use of laboratory animals and those of the Italian Ministry of Health (DL 116/92), and they were approved by the Institutional Animal Care and Use Committee at our institution.

### Cell cultures and treatments

Rat primary astroglial cultures were obtained from newborn Sprague-Dawley rats (1 or 2 days old) according to the procedure described by Vairano et al. [24]. Brain homogenates were mechanically processed to obtain single cells that were seeded in 75-cm^2 ^flasks at a density of 3 × 10^6 ^cells/flask with 15 ml of culture medium (DMEM, 5% inactivated foetal bovine serum, 100 IU/ml penicillin and 100 μg/ml streptomycin; all from Sigma-Aldrich, Milan, Italy) and incubated at 37°C in a humidified atmosphere containing 5% CO_2_. The culture medium was replaced after 24 hours and again twice weekly until astrocytes were grown to form a monolayer firmly attached to the bottom of the flask (7 or 8 days after dissection). At cell confluence, flasks were vigorously shaken to separate astrocytes (which remained adherent in the bottom of the flasks) from microglia and oligodendrocytes (which floated on the supernatant). The same process was repeated after about 1 week of culture. Collected astrocytes were seeded onto 10-cm-diameter Petri dishes at a density of 1 × 10^6 ^cells/dish. The purity of the cells in culture was tested with monoclonal anti-glial fibrillary acidic protein (GFAP), and only cultures with more than 95% GFAP-positive cells were used for the experiments. The 5% of nonastrocyte cells were microglia and oligodendrocytes.

Cultures of rat primary neurons were prepared from embryonic day 18 Sprague-Dawley rats according to the method described by Antonelli et al. [25]. Pregnant female Sprague-Dawley rats were killed by CO_2_. The uterus containing the embryos was removed from the adult rat. The embryos were decapitated, and the brain was removed from the cranium. Removed cortices were dissected free of meninges and dissociated in 0.025% (wt/vol) trypsin. The tissue fragments were dissociated mechanically through a glass Pasteur pipette. The cells were cultured in Neurobasal Medium supplemented with 0.1 mM glutamine, 10 μg/ml gentamicin and 2% B27 (all purchased from Invitrogen/Life Technologies, Monza, Italy). Cells were grown at 37°C in a humidified atmosphere containing 5% CO_2_. The cultures were left to grow for 1 week to reach a stage similar to the cells prepared from newborn rat pups.

Mature astrocytes and neurons were counted by direct microscopic counting using trypan blue (Sigma-Aldrich) staining in a Bürker chamber and co-cultured at a ratio of approximately 10:1. Cells pelleted and resuspended in Neurobasal Medium supplemented with 0.1 mM glutamine, 10 μg/ml gentamicin and 2% B27. Next these mixed neuroglia co-cultures were plated on glass slide chambers coated with poly-D-lysine (BD Biosciences, Buccinasco, Italy) at a density of 2.5 × 10^4 ^cells/chamber and cultured at 37°C in a humidified atmosphere containing 5% CO_2 _for 24 hours before treatment.

Mixed neuroglial co-cultures were treated with 1 μg/ml Aβ_1-42 _(Tocris Bioscience, Bristol, UK) in the presence or absence of the following substances: PEA (0.1 μM), MK886 (3 μM), the selective PPARα antagonist, and GW9662 (9 nM), the selective PPARγ antagonist (all purchased from Tocris Bioscience). After 24 hours of treatment, cells were processed for analyses. The concentration of the substances was chosen according to our previous results [23]. No significant variation from control was observed when PEA, MK886 or GW9662 was given alone (data not shown).

### Preparation of organotypic cultures and treatments

Organotypic hippocampal slice cultures were prepared according to the method described by Pellegrini-Giampietro et al. [26]. Briefly, 7-day-old Sprague-Dawley rats were killed by decapitation, and the extracted brains were transversely cut using a vibratome (Microm HM 650 V; Microm International GmbH Part of Thermo Fisher Scientific Walldorf, Germany) to obtain 400-μm coronal sections containing the hippocampi. These slides were placed onto semiporous inserts (4 μm in diameter; Millipore, Vimodrone, Italy) and cultured in 6-cm-diameter Petri dishes with 1.2 ml of DMEM supplemented with 25% Hank's Balanced Salt Solution (Invitrogen/Life Technologies), 25% heat-inactivated horse serum (Sigma-Aldrich), 20 mM 4-(2-hydroxyethyl)-1-piperazineethanesulfonic acid and 1.5% penicillin-streptomycin at 37°C in a humidified atmosphere containing 5% CO_2_. The culture medium was refreshed upon necessity.

On day 21 of culturing, organotypic hippocampal slide cultures were treated with 1 μg/ml Aβ_1-42 _in the presence or absence of the following substances: PEA (0.1 μM), MK886 (3 μM) or GW9662 (9 nM), with the latter two being selective PPARα and PPARγ antagonists, respectively. Twenty four hours after the treatments, sections were washed twice with 1 × PBS and fixed overnight at 4°C with 4% paraformaldehyde in 1 × PBS, then slices were gently removed from inserts and processed for morphological and immunofluorescence experiments.

### Nissl staining

Mounted sections were sequentially dipped in different alcohol solutions of decreasing concentration to remove lipids from the tissue, then they were stained with 2% cresyl violet solution for 5 minutes and finally brain sections were dehydrated with a series of baths of increasing alcohol concentrations. Sections were observed through a microscope (Nikon Eclipse 80i; Nikon Instruments Europe, Kingston upon Thames, UK). Corresponding pictures were captured at 2 × magnification using a high-resolution digital camera (Nikon Digital Sight DS-U1; Nikon Instruments Europe) and analyzed using NIS-Elements software (Nikon Instruments Europe).

### Immunofluorescence

Both mixed neuroglial and glass-mounted organotypic cultures were washed with 1 × PBS and fixed with 4% paraformaldehyde in 1 × PBS. Afterwards samples were blocked in 10% albumin bovine serum 0.1% Triton-PBS solution for 90 minutes, then they were incubated for 1 hour with a 10% albumin bovine serum/0.1% Triton-PBS solution containing the following antibodies: anti-GFAP (1:500 dilution; Abcam plc, Cambridge, UK) and anti-microtubule-associated protein 2 (MAP2) (1:200, Novus Biologicals, Milan, Italy). Finally, samples were incubated for 1 hour in the dark with the proper secondary antibodies: fluorescein isothiocyanate-conjugated anti-rabbit at 1:100 dilution or Texas Red-conjugated anti-mouse at 1:64 dilution (both from Abcam plc), respectively. Nuclei were stained with Hoechst at 1:5,000 dilution (Sigma-Aldrich, St. Louis, MO, USA) added to the secondary antibody solution.

Pictures were taken using a camera (Nikon Digital Sight DS-U1) connected to a microscope (Nikon Eclipse 80i; Nikon Instruments Europe) provided with the proper fluorescence filters. Slides were analyzed with a microscope (Nikon Eclipse 80i), and images were captured at 10 × and 20 × magnification with a high-resolution digital camera (Nikon Digital Sight DS-U1). Analysis of immunopositive cells was performed using a specific digital system (NIS-Element Basic Research version 2.30 software).

### Statistical analysis

Results are expressed as means ± SEM of the experiments. Statistical analysis was performed using parametric one-way analysis of variance, and multiple comparisons were performed using the Bonferroni test with the GraphPad InStat statistical software program (GraphPad Software, La Jolla, CA, USA). *P *< 0.05 was considered significant.

## Results and discussion

The classical amyloid cascade hypothesis claims that an imbalance between the production and degradation or clearance of Aβ in the brain represents the initiating event in AD neuropathology, leading to synaptic damage and neuronal death. In the current investigation, experiments were performed utilizing models that, in many respects, although not fully, reflect conditions that occur in the brain in AD. The results of the present study demonstrate that PEA treatment causes a significant, marked reduction of astrocyte activation and a parallel neuronal protection in both mixed neuroglial and organotypic hippocampal cultures.

**Figure 1:**
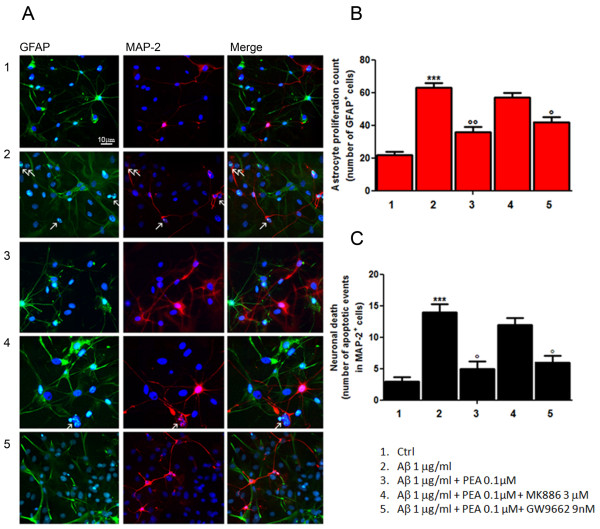
**PEA inhibits astroglial proliferation and reduces neuronal loss in mixed neuroglia co-cultures exposed to Aβ**. Aβ-challenged (1 μg/ml) astrocyte/neuron mixed cultures were treated with PEA (0.1 μM) in the presence of the selective PPARγ antagonist (GW9662, 9 nM) or the selective PPARα antagonist (MK886, 3 μM). After 24 hours of treatment, cells were processed for analyses. **(A) **Immunofluorescence photomicrographs showing the effect of the treatments on astrocyte proliferation and neuronal loss, as determined by immunostaining for GFAP (green) and MAP2 (red), respectively. Arrows indicate chromatin condensation in nuclei stained with Hoechst dye (blue) as markers of apoptotic events. Scale bar: 10 μm. **(B) **Relative quantification of GFAP-positive cell number as a count of astrocyte proliferation. **(C) **Apoptotic events detected on MAP2-expressing cells as an indication of neuronal death. For (B) and (C), the average value was determined by counting cells in at least nine microscopic fields for each treatment. Results are presented as means ± SEM of three separate experiments. Statistical analysis was performed using parametric one-way analysis of variance, and multiple comparisons were performed using the Bonferroni test. ***P < 0.001 vs. unstimulated cells, ^∘∘^*P *< 0.01 and ^∘^*P *< 0.05 vs. Aβ-stimulated cultures.

We have previously demonstrated that PEA strongly downregulates reactive gliosis by reducing proinflammatory molecules and cytokine release through the inhibition of NF-κB in rat astrocytes [23]. The present findings extend our knowledge of PEA pharmacology. In particular, they indicate that such modulation of astrocyte function accounts for a rebound neuroprotection. Indeed, data from mixed neuroglial co-cultures indicate that PEA treatment results in a massive reduction (in comparison with the Aβ group) in astrocyte number, as shown by the reduction in the GFAP-immunopositive cells (Figures 1A and 1B). Consequently, such an effect is accompanied by a significant decrease in the number of apoptotic nuclei in MAP2-positive neurons induced by Aβ challenge (Figures 1A and 1C). Photomicrographs indicate that the PEA antigliosis and neuroprotective effects are inescapably due to PPARα involvement because MK886, the selective PPARα antagonist, almost completely abolishes the PEA effects, whereas GW9662, the selective PPARγ antagonist, does not show any detectable influence.

**Figure 2:**
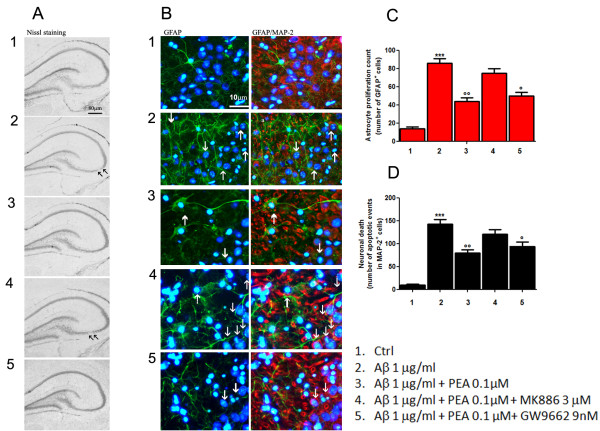
**PEA decreases astrocyte activation in organotypic cultures of rat hippocampi and rescues neuronal CA3 damage caused by Aβ challenge**. Aβ-challenged (1 μg/ml) slices of rat hippocampi were treated for 24 hours with PEA (0.1 μM) in the presence of the selective PPARγ antagonist (GW9662, 9 nM) or the selective PPARα antagonist (MK886, 3 μM). **(A) **Nissl staining showing the effect of treatment on the morphology of organotypic hippocampal slices. Scale bar: 80 μm. Aβ caused a marked neuronal loss, mainly in the CA3 region of hippocampus, as highlighted by arrows. PEA was able to reverse this effect. **(B) **Representative photomicrographs of the CA3 region showing the results of immunofluorescence experiments aimed at investigating the effect of treatments on astrocyte activation and neuronal loss, as determined by immunostaining for GFAP (green) and MAP2 (red) alone or merged, respectively. Nuclei were stained with Hoechst (blue). Scale bar: 10 μm. Arrows in the photomicrographs indicate astrocyte infiltration events and apoptotic condensation in the nuclei of adjacent neurons. **(C) **Relative quantification of GFAP-positive cell number as a count of astrocyte proliferation. **(D) **Apoptotic events detected on MAP2-expressing cells as an indication of neuronal death. For (C) and (D), the average value was determined by counting cells in at least five microscopic fields for each treatment. Results are presented as means ± SEM of four separate experiments. At least four slices from each experimental group were observed for each experiment. Statistical analysis was performed using parametric one-way analysis of variance, and multiple comparisons were performed using the Bonferroni test. ***P < 0.001 and *P < 0.05 vs. control; ^∘∘^*P *< 0.01 and ^∘^*P *< 0.05 vs. Aβ-challenge slices.

General overview of Nissl-stained hippocampi indicates that Aβ treatment results in a depletion of CA3 pyramidal neurons compared to controls. PEA treatment rescues the integrity of this area, and, in agreement with the above-described in vitro results, its neuroprotective effect appears to be dependent on PPARα interaction (Figure 2A).

Furthermore, immunofluorescence analysis of the CA3 area of the same hippocampi reveals a marked activation and an infiltration of astrocytes after Aβ treatment (Figure 2). In fact, we observed an evident increase in the size and number of GFAP-immunopositive cells that paralleled a higher number of apoptotic nuclei in MAP2-positive neurons (Figures 2B through 2D). Such effects were counteracted by treatment with PEA, and we also observed in these experiments that its action was strongly dependent on the selective activation of PPARα (Figure 2B).

In conclusion, our data provide evidence that PEA blunts reactive gliosis and subsequently prevents neuronal damage in models of Aβ neurotoxicity. These observed effects are strictly dependent on the activation of PPARα. In addition, although some authors have hypothesized that a number of pharmacological actions of PEA could be mediated by modifications occurring in the endocannabinoid system, no significant changes were observed in either 2-arachidonoylglycerol or anandamide levels in our experimental conditions (data not shown). This assumption further supports a key role for PPARα as a molecular target at which PEA acts to mitigate the toxic effects induced by Aβ.

The relevance of these results resides in the hypothesis that pharmacological attenuation of excessive and prolonged reactive gliosis may serve as an innovative strategy for therapies aimed at ameliorating the course of AD. There is an urgent need for new molecules that will affect different pathological pathways, all converging on progressive neurological decline. Our data suggest that PEA is capable of profoundly reducing reactive astrogliosis and of guaranteeing neuronal protection in Aβ-induced neuroinflammatory and neurodegenerative events.

## Abbreviations

Aβ: β-amyloid; AD: Alzheimer's disease; CNS: central nervous system; DMEM: Dulbecco's modified Eagle's medium; GFAP: glial fibrillary acidic protein; NF-κB: nuclear factor κB; NFT: neurofibrillary tangle; PBS: phosphate-buffered saline; PEA: palmitoylethanolamide; PPAR: peroxisome proliferator-activated receptor; SP: senile plaque.

## Competing interests

The authors declare that they have no competing interests.

## Authors' contributions

CS conceived the study, carried out the experiments, drafted and revised the manuscript. MV carried out the immunofluorescence analysis. CS conducted the experiments in neuronal cultures. GE participated in the design of the study and prepared the figures. MRC performed the statistical analysis and helped to draft the manuscript. LS participated in defining the experimental design of the study, contributed to the interpretation of results and reviewed the manuscript. All the authors read and approved the final manuscript.
